# Awareness of deleterious oral habits and knowledge of habit breaking appliance among parents in Alkharj - A cross sectional study

**DOI:** 10.12688/f1000research.157200.1

**Published:** 2024-10-29

**Authors:** Sultan Abdulrahman Almalki, Inderjit Murugendrappa Gowdar, Saeed N Asiri, Praveen Jodalli, Bashar Ayed Alanazi, Osama abdullah Alqahtani, Fahad Radhi Alanazi

**Affiliations:** 1 Department of Preventive Dental Sciences, College of Dentistry, Prince Sattam bin Abdul Aziz University, Alkharj, 11942, Saudi Arabia; 2 Department of Pediatric Dentistry, College of Dentistry, Prince Sattam bin Abdul Aziz University, Alkharj, 11942, Saudi Arabia; 3 Public Health Dentistry, Manipal College of Dental Sciences Mangalore, Manipal Academy of Higher Education, Manipal, Karnataka, 576104, India

**Keywords:** oral appliance, oral health, dental appliance, habits

## Abstract

**Background:**

Actions that are repetitive and being practiced automatically are called habits. Oral habits can be classified as normal or deleterious. Oral health education starts from footprints of awareness. Growing children require appropriate guidance for healthy growth and maintenance of their teeth, which makes the parent an active participant in the prevention and treatment of malocclusion and thus is majorly affected by their knowledge and attitude regarding various deleterious oral habits and habit breaking appliances.

**Aim:**

To assess and compare awareness of deleterious oral habits and knowledge of habit breaking appliance, among parents at Alkharj Saudi Arabia.

**Methodology:**

Study assessed awareness of deleterious oral habits and knowledge of habit breaking appliances among parents in Alkharj, Saudi Arabia.

**Results:**

**Conclusion:**

Majority of the parents were aware of different deleterious oral habits but not aware of the dimensions of interventions like types of appliances, duration of use, etc. without knowing mentioned facts, in future which leads to development of malocclusion which probably requires orthodontic, surgical or prosthetic intervention correcting the outcomes of oral deleterious habits.

## Introduction

Actions that are repetitive and being practiced automatically are called habits. Oral habits are learned patterns of muscle contraction and have a very complex nature. ^1^ Oral habits can be classified as normal or deleterious. Nasal breathing, chewing, and swallowing are regarded as physiological and functional habits, since they contribute to the establishment of normal occlusion, favoring the harmonic facial growth without deviations. ^2^ However, habits which influences dentofacial structures and results in malocclusion such as nail biting, mouth breathing, thumb sucking, finger sucking, tongue thrusting, lip biting, or lip sucking, bruxism are called deleterious or adverse oral habits. ^3, 4^ These deleterious habits have regular tendency and is hard to give up. These habits are mostly seen in the early childhood and mixed dentition stages. ^5^

Malocclusion is the misalignment of teeth resulting in esthetic and functional alterations. The common causes of malocclusion include genetic, environmental, systemic causes, and harmful oral habits. ^6^ Hence, oral habits are the primary cause of dentofacial deformities in children, require immediate attention for prevention. Knowledge and awareness are essential prerequisites for changes in behavior related to health and disease anticipation. ^7^

Nowadays the focus in dentistry is changing towards prevention of disease rather than its treatment and the public’s role has changed from passive recipient to active participant in prevention. ^8^ Children with extracted anterior teeth along with the habit of thumb sucking require attention commonly for aesthetics, function, and space maintenance but also must be given an appliance for habit breaking. The line of treatment for these habits includes the removal of the etiology, retraining exercises, and the use of mechanical restraining appliances. ^9^

Parents are role models for their children. They can play an important role in developing healthy oral habits in children. ^10^ Oral health education starts from footprints of awareness. Growing children require appropriate guidance for healthy growth and maintenance of their teeth, which makes the parent an active participant in the prevention and treatment of malocclusion and thus is majorly affected by their knowledge and attitude regarding various deleterious oral habits and habit breaking appliances. No data are available regarding parental awareness of deleterious oral habits and habit breaking appliances among Saudi parents. Hence, the present study was conducted to assess the parental knowledge and awareness of deleterious oral habits and habit breaking appliance among Alkharj parents.

## Methods

The survey is designed, analyzed and interpreted according the STROBE checklist.

### Study design and population

An observational, descriptive cross-sectional survey was conducted on adult population to assess the awareness oof deleterious oral habits and their knowledge on habit breaking appliances in Alkharj, Saudi Arabia.

### Study proforma

Data were collected using a self-designed structured questionnaire comprising 12 questions, divided into three sections. First section gathered basic demographic details of the participants; the second section included four questions aimed at assessing participants’ awareness of harmful oral habits. The final section contained six questions focused on participants’ knowledge regarding malocclusion and various appliances used to correct these issues.

### Validation of questionnaire

The questions were translated into Arabic and subsequently back-translated into English by language experts proficient in both languages. This back-translation method was employed to verify the translation’s validity and ensure linguistic equivalence. Content validity was assessed by two subject matter experts in orthodontics, and the Content Validity Index (CVI) for the entire scale was calculated. A satisfactory level of agreement was achieved, with a CVI of 0.87 among the panellists.

A pilot survey was conducted, during which the questionnaire was administered randomly to ten parents. After two days, the same questionnaire was readministered to these parents to evaluate reliability using the test-retest method. The computed reliability value was 0.89. Additionally, Cronbach’s α was applied to assess internal consistency, yielding a value of 0.84, indicating a high level of correlation.

### Date collection

Parents visiting the screening area of the College of Dentistry in Alkharj, along with their children seeking treatment, were approached to participate in the survey after meeting the eligibility criteria. A scan code for the questionnaire was sent to their Messenger, WhatsApp, Snapchat, or email. The questionnaire included multiple sections: a description of the survey, a voluntary written consent form, the survey questions, and a thank-you note. To enhance the response rate, three reminders were sent to the parents. A questionnaire was sent to 400 parents, of whom 376 completed it, resulting in a response rate of 89%. The primary reason for non-response was reported as a lack of time.

**Validation of questionnaire:** Based on the detailed analysis of literature, structured questionnaire was framed with the assistance and guidance of panel of academic experts to achieve validated and relevant questionnaire. To validate the questionnaire, questions were prepared in English language initially and translated to Arabic language & it was back translated again to English.

### Sample size

Sample size was calculated by using the formula

$$N=\frac{Z^{2}\mathrm{pq}}{d^{2}}$$

Where,

Z = 1.96 (Confidence Level or 5% level of significance)

p = awareness level of deleterious habits at 60% = 0.60

q=(1–p)100−60=40%=0.40

d = Precision limit or proportion of sampling error which is usually 5% confidence limit. = 0.05

By substituting the values, sample size of 368 was found to be sufficient.

**Questionnaire:** An anonymous, objective type questionnaire was requested to fill by parents without providing any information. Questionnaire had 3 parts Personal information (gender, age, place and educational status), awareness about deleterious habits and knowledge of habit breaking appliance. Unmarried people were not included in the study only parents who have children above 7 years and parents who were residing in Alkharj were included.

**Method:** Questionnaire scan code was prepared and was asked to scan when parents came with children for treatment at screening area of College of Dentistry. Parents were asked to send the link to friends and groups in Alkharj through cross platform messengers WhatsApp, snap chat and email which also had a consent form and explanation about the study. A total of 400 questionnaires were sent out of which 376 returned giving a response rate of 89% out of which 7 questionnaire were incomplete and were excluded hence a total of 369 responses were considered.

**Statistical analysis:** Descriptive statistics were computed and data were analyzed statistically using chi square test with 95% confidence interval and p value less than 0.05 was considered as statistically significant.

## Results

There are total of 369 participants in the study out of which 163 (44.2%) are males and 206 (55.8%) are females distribution of study participants according to age is presented in Table 1.

**Table 1.** Distribution of study subjects according to gender and age.

Gender	Age	Frequency	Percent	Total
Males	< 30 years	45	27.60	163 (44.2)
	30 – 50 years	99	60.73	
	> 50 years	19	11.65	
Females	< 30 years	17	8.25	206 (55.8)
	30 – 50 years	124	60.19	
	> 50 years	65	31.55	
Total		369	100.0	369

Table 2 shows awareness of study participants about various deleterious habits. 80% of study participants were aware about thumb sucking habit and mouth breathing habits. Whereas 75.6% of study participants were aware about bruxism and only 34.1% of study participants are aware about tongue thrusting habit.

**Table 2.** Awareness of study subjects about deleterious oral habits.

Habits	Response	Frequency	Percentage
Are you aware of aware of thumb sucking habit	Yes	298	80.8
	No	71	19.2
Are you aware of aware of mouth breathing habit	Yes	297	80.5
	No	72	19.5
Are you aware of tongue thrusting habit	Yes	126	34.1
	No	273	65.9
Are you aware of bruxism?	Yes	279	75.6
	No	90	24.4

Table 3 shows response of study participants about malocclusion and habit breaking appliance. 46.9% of study subjects are not sure whether adverse habits results in malocclusion or not. 55.3% of participants were not sure that deleterious habits can be treated with appliances are not. 84.0% of participants are not sure about the duration of usage of an habit breaking appliance. 68.9% of study subjects are not sure of the usage of habit breaking appliance in preventing malocclusion. 52.8% of subjects told that Adverse habits can be due to feeling of insecurity and anxiety. 66.1% of subjects think that psychological counseling will benefit in breaking adverse oral habits.

**Table 3.** Response of study subjects about malocclusion and habit breaking appliances.

Habits	Response	Frequency	Percentage
Oral adverse habits can lead to malocclusion	Yes	188	50.9
	No	8	2.2
	Not sure	173	46.9
Adverse oral habits can be treated with habit breaking appliances	Yes	161	43.6
	No	4	1.1
	Not sure	204	55.3
Habit breaking appliance should not be used for more than six months	Yes	59	16.0
	No	0	0.0
	Not sure	310	84.0
Habit breaking appliance helps in preventing malocclusion	Yes	109	29.5
	No	6	1.6
	Not sure	254	68.9
Adverse habits can be due to feeling of insecurity and anxiety	Yes	195	52.8
	No	40	10.8
	Not sure	134	36.3
Can psychological counseling benefit in breaking adverse oral habits	Yes	244	66.1
	No	03	8
	Not sure	122	33.1

Table 4 shows comparison of responses of study participants according to gender and age group. Age group showed statistically significant differences with all the questions evaluated whereas according to gender few of the responses were statistically significant as shown in the table.

**Table 4.** Response of study subjects about malocclusion and habit breaking appliances.

Habits	Gender	Age
Are you aware of aware of thumb sucking habit	χ ^2^ = 13.15 p < 0.05 S	χ ^2^ = 24.69 p < 0.05 S
Are you aware of aware of mouth breathing habit	χ ^2^ = 18.35 p < 0.05 S	χ ^2^ = 22.04 p < 0.05 S
Are you aware of tongue thrusting habit	χ ^2^ = 1.39 p = 0.269 NS	χ ^2^ = 11.10 p = 0.04 S
Are you aware of bruxism?	χ ^2^ = 0.300 p = 584 NS	χ ^2^ = 6.14 p = 0.035 S
Oral adverse habits can lead to malocclusion	χ ^2^ = 18.27 p < 0.05 S	χ ^2^ = 19.65 p = 0.01 S
Adverse oral habits can be treated with habit breaking appliances	χ ^2^ = 8.871 p = 0.12 S	χ ^2^ = 14.08 p = 0.007 S
Habit breaking appliance should not be used for more than six months	χ ^2^ = 11.659 p = 0.01 S	χ ^2^ = 9.40 p = 0.012 S
Habit breaking appliance helps in preventing malocclusion	χ ^2^ = 6.775 p = 0.034	χ ^2^ = 45.32 p < 0.05 S
Adverse habits can be due to feeling of insecurity and anxiety	χ ^2^ = 2.65 p = 0.265 NS	χ ^2^ = 34.53 p < 0.05 S
Can psychological counseling benefit in breaking adverse oral habits	χ ^2^ = 3.845 p = 0.146 NS	χ ^2^ = 35.39 p < 0.05 S

## Discussion

An automatic act adapted by practice and performed with repetition is called as habit. These habits can be deleterious and can be the major contributing act leads to the development of dental malocclusion. ^11^ Actions includes sucking of digits, mouth breathing, habits associated with pacifiers and bottle use, lip biting and suckling habits, tongue thrusting, teeth clenching and if these acts, performed in repetition manifests as an automated act to become oral deleterious habits. ^12^ Among all, thumb suckling and tongue thrusting habits are most commonly prevailed acts giving rise to variety of oro-facial malformations. ^13^

Children practicing the habits are generally unaware of the deleterious habits and their long-term impact of any habit and parents should take vital role by acting with timely intervention leading to the cessation of habit. ^9^ Present study attempts to assess the knowledge and awareness of habits and habit breaking appliances among Alkharj parents Saudi Arabia.

Among 369 participants in the study, 163 were males and 206 were females and majority of the participants were middle aged individuals aged between 30 to 50 years. Majority of the study participants were aware of thumb sucking habit as it is highly prevalent and same observations prevailed with mouth breathing and bruxism but surprisingly only 34% among the participants were aware of the existence of tongue thrusting as a habit which is noted to be prevalent among different population and current study findings is in accordance with findings of Prasanna et al. ^9^

On assessment of awareness of habits and habit breaking appliances only 50% of the participants were aware of adverse habits and their manifestations and nearly 47 % of the participants were not sure of the effects and outcomes of the deleterious habits which can adversely affects the maxillofacial development and this mirrored with the findings of Hedge et al. ^14^ and further, 55% of the participants were not sure of interventional aspects involving habit breaking appliances. Assessing the observations on duration of administration of treatment involving habit breaking appliances, nearly 70% of the participants were totally unaware of the concepts and trends of appliances and similar findings is noted by Chonat et al. ^15^

On observations on psychological dimensions of the oral deleterious habits, majority of the participants were able to relate the existence of secondary factors like insecurity and anxiety as it is a established fact that habits will have psychological association and majority of the participants also believe psychological counseling benefits in breaking adverse oral habits and contrasting results been noted by Gangurde et al, probably the difference is accounted by selected study population. ^16^

On summarizing the current study results majority of the parents were aware of different deleterious adverse oral habits but not aware of the dimensions of interventions like types of appliances, duration of use etc without knowing mentioned facts in future which leads to development of malocclusion which probably requires orthodontic, surgical or prosthetic intervention correcting the outcomes of oral deleterious habits. To spread awareness among parents different awareness programmes involving different strata of the society should be conducted by various government and non-government organizations to impact prevention. Those programmes may include awareness camps, descriptive digital demonstrations, community lectures, publicizing on social media, conceptual facts published in newspapers, displaying posters in public platforms and also timely updating information on the same to prevent long term impact of oral deleterious habits.

## Ethics and consent

The study protocol received approval from the Institutional Review Board (IRB) of Prince Sattam Bin Abdul Aziz University, under approval number SCBR-198/2023 on 19/12/2023 Voluntary written informed consent was obtained from all participants using the electronic informed consent system prior to accessing the online survey link. The consent form clearly described the purpose of the survey and amount of time required to complete the questionnaire in simple and understandable language. Participants were informed that they could withdraw from the survey at any time without penalty and that their personal information would be kept confidential.

## Data Availability

Awareness of deleterious oral habits and knowledge of habit breaking appliance among parents in Alkharj - A cross sectional study. figshare. https://doi.org/10.6084/m9.figshare.27111499. ^17^ This project contains the following underlying data:
1. Ortho excel sheet.xlsx Data are available under the terms of the Creative Commons Zero “No rights reserved” data waiver (CC0 1.0 Public domain dedication). Awareness of deleterious oral habits and knowledge of habit breaking appliance among parents in Alkharj - A cross sectional study. figshare. Dataset 2024. https://doi.org/10.6084/m9.figshare.27111499. ^17^ This project contains the following extended data:
1. Ortho questionnaire.dox 2. Questionnaire translated.dox 3. STROBE ckecklist.doc Data are available under the terms of the Creative Commons Zero “No rights reserved” data waiver (CC0 1.0 Public domain dedication).
