# Supplementary material for: Awareness of deleterious oral habits and knowledge of habit breaking appliance among parents in Alkharj - A cross sectional study
Source: F1000Res. 2024 Oct 29;13:1295. [Version 1] doi: 10.12688/f1000research.157200.1 (PMC11584452; doi:10.12688/f1000research.157200.1)
Supplement: Peer review history

# Reviewer report: Reviewer response for version 1

**DOI:** 10.5256/f1000research.172618.r340596

**Published:** 2024-12-05

**Authors:** Sana Bint Aziz

**Affiliations:** 1 Manav Rachna Dental College, Faridabad, Haryana, India

The manuscript titled " Awareness of deleterious oral habits and knowledge of habit breaking appliance among parents in Alkharj - A cross sectional study" is interesting as awareness of deleterious oral habits is important for the prevention and timely management. The manuscript is written well, however there are certain areas that need some revisions.

 Abstract:

 No result is provided. Please include in brief the findings of the study in the result section of the abstract.

 Methods:

 Check spellings in line 'to assess the awareness oof deleterious oral habits" and " Date collection"

 Please provide a reference for the Content Validity Index (CVI) and include it in the manuscript.

 The methods section needs to be reframed to clearly state as to how many times the questionnaire was validated and how many questionnaires were prepared. Certain sub-headings like validation of questionnaire, data collection,  method, and questionnaire are overlapping and repetitive in nature, resulting in confusion about the no. of questionnaires used in the study. For instance, If a pilot study was conducted before the main survey then it can be explained in detail in the beginning only under a sub-heading of Pilot study.

 Sample Size - Please mention whether the sample size calculation was done a priori (before the study) or later.

 Results

 Please break the sentence in two, or join the two sentences using a conjunction such as 'and'... "There are total of 369 participants in the study out of which 163 (44.2%) are males and 206 (55.8%) are fe males distribution of study participants according to age is presented in Table 1."

 Discussion

 Limitations of the study has not been included. Please include, if any.

 Also, a short conclusion at the end of the manuscript is needed.

Is the work clearly and accurately presented and does it cite the current literature?

Yes

If applicable, is the statistical analysis and its interpretation appropriate?

Yes

Are all the source data underlying the results available to ensure full reproducibility?

Yes

Is the study design appropriate and is the work technically sound?

Yes

Are the conclusions drawn adequately supported by the results?

Yes

Are sufficient details of methods and analysis provided to allow replication by others?

Yes

Reviewer Expertise:

Orthodontics & Dentofacial Orthopedics

I confirm that I have read this submission and believe that I have an appropriate level of expertise to confirm that it is of an acceptable scientific standard, however I have significant reservations, as outlined above.

# Reviewer report: Reviewer response for version 1

**DOI:** 10.5256/f1000research.172618.r340595

**Published:** 2024-11-22

**Authors:** Tanushree Dalvi

**Affiliations:** 1 Bharati Vidyapeeth Dental College and Hospital, Sangli, Maharashtra, India

The manuscript is well structured and drafted according to the STROBE guidelines. Title and abstract is informative. The introduction highlights the need for the study with aim. The authors have explained in detail about the validation of the instrument of data collection, the study design and the scientific estimation of sample size with formula. The method of data collection is explained in depth to allow replication. The tests for statistical analysis have been specified according to the type of data obtained. The results are well tabulated and the characteristics of study participants have been mentioned. The authors have summarised the key results in discussion and also cited studies with similar findings. Recommendation is provided for executing similar research for future studies. Ethical approval is obtained and appropriate referne ID id mentioned along with the consent process in detail.

Is the work clearly and accurately presented and does it cite the current literature?

Yes

If applicable, is the statistical analysis and its interpretation appropriate?

Yes

Are all the source data underlying the results available to ensure full reproducibility?

Yes

Is the study design appropriate and is the work technically sound?

Yes

Are the conclusions drawn adequately supported by the results?

Yes

Are sufficient details of methods and analysis provided to allow replication by others?

Yes

Reviewer Expertise:

Epidemiological investigations, Health Education, cross-sectional studies, Oral Health

I confirm that I have read this submission and believe that I have an appropriate level of expertise to confirm that it is of an acceptable scientific standard.

# Reviewer report: Reviewer response for version 1

**DOI:** 10.5256/f1000research.172618.r340591

**Published:** 2024-11-22

**Authors:** Omnia Abdallah

**Affiliations:** 1 Misr International University, Cairo, Cairo Governorate, Egypt

highly informative, please proceed with indexing

 Awareness of deleterious oral habits and knowledge of habit breaking appliance among parents in Alkharj - A cross sectional study

 The authors have indicated the gap between what science have indicated and the parents knowledge and ability to convey this information to their children

 minor revisions

 1- the authors need to share the sections of the survey if not the whole one as supplementary material 

 2- the authors need to add a further paragraph to the discussion as their own conclusion and future implementation of such results.

Is the work clearly and accurately presented and does it cite the current literature?

Yes

If applicable, is the statistical analysis and its interpretation appropriate?

Yes

Are all the source data underlying the results available to ensure full reproducibility?

Yes

Is the study design appropriate and is the work technically sound?

Yes

Are the conclusions drawn adequately supported by the results?

Yes

Are sufficient details of methods and analysis provided to allow replication by others?

Yes

Reviewer Expertise:

microbiology, biotechnology, nanotechnology

I confirm that I have read this submission and believe that I have an appropriate level of expertise to confirm that it is of an acceptable scientific standard.

# Reviewer report: Reviewer response for version 1

**DOI:** 10.5256/f1000research.172618.r340598

**Published:** 2024-11-19

**Authors:** Thippeswamy HM

**Affiliations:** 1 JSS Academy of Higher Education and Research, Mysuru, Karnataka, India

The study found to be good. introduction explained with seminar approach and content found to be good. Study methodology presented well. Administration of questionnaire to the parents explained very well. validation, sample size done well. overall methodology seems to be good. Results presented tabular form. Inferential statistics values would have mentioned then and there, instead of making separate table 4. Discussion and conclusion written well.

Is the work clearly and accurately presented and does it cite the current literature?

Yes

If applicable, is the statistical analysis and its interpretation appropriate?

Yes

Are all the source data underlying the results available to ensure full reproducibility?

Yes

Is the study design appropriate and is the work technically sound?

Yes

Are the conclusions drawn adequately supported by the results?

Yes

Are sufficient details of methods and analysis provided to allow replication by others?

Yes

Reviewer Expertise:

Public Health dentist

I confirm that I have read this submission and believe that I have an appropriate level of expertise to confirm that it is of an acceptable scientific standard.
